# Effects of carbohydrate counting training versus standard nutritional and medical therapy on glycemic control and sarcopenia in type 2 diabetes: an open-label randomized controlled trial

**DOI:** 10.1186/s13098-026-02258-1

**Published:** 2026-08-18

**Authors:** Funda Gargaci Tuncer, Indrani Kalkan, Ozden Ezgi Uner, Serkan Elarslan

**Affiliations:** 1 Graduate School of Health Sciences, Department of Nutrition and Dietetics, Istanbul Medipol University, Kavacik South Campus, Istanbul, 34810 Turkey; 2 Faculty of Health Sciences, Department of Nutrition and Dietetics, Istanbul Medipol University, Istanbul, Turkey; 3 Istanbul Fatih Sultan Mehmet Training and Research Hospital, Faculty of Medicine, Department of Family Medicine, University of Health Sciences, Istanbul, Turkey; 4 Istanbul Fatih Sultan Mehmet Training and Research Hospital, Faculty of Medicine, Department of Internal Medicine, University of Health Sciences, Istanbul, Turkey

**Keywords:** Diabetes, Carbohydrate, Carbohydrate counting, Glycemic control, Insulin

## Abstract

**Introduction:**

Carbohydrate counting training is recommended for patients with type 1 diabetes as it has been proved to be effective in providing glycemic control. However, there is limited evidence of this training on glycemic control in patients with type 2 diabetes. Furthermore, there is limited knowledge on presence of sarcopenia in this group.

**Objective:**

To examine sarcopenia in patients with type 2 diabetes receiving intensive insulin therapy and evaluate the effect of carbohydrate counting training on clinical findings related to diabetes and sarcopenia.

**Method:**

A 24-week randomized controlled trial was conducted on 58 individuals with type 2 diabetes receiving intensive insulin therapy (59.81 ± 10.24 years, HbA1c level: 9.42 ± 1.73%). Participants were randomly divided into two groups: control (standard medical and nutrition treatment) and training (advanced carbohydrates counting training in addition to standard treatment).

**Results:**

Daily prandial insulin doses decreased in the training group compared to baseline (*p* = 0.034). The frequency of hypoglycemia and hyperglycemia decreased significantly (*p* = 0.007 and *p* = 0.001, respectively). HbA1c levels in the training group decreased by 1.04 ± 1.01% and 1.56 ± 1.35% at weeks 12 and 24, respectively. Although a significant time-by-group interaction was observed, post-hoc comparisons were not significant after Bonferroni correction. A significant decrease was found in body weight, Body Mass Index (BMI) and muscle mass in the training group. Hand grip test conducted on all participants indicated 19.0% to have low muscle strength.

**Conclusion:**

Advanced carbohydrate counting training was associated with improvements in glycemic parameters in individuals with type 2 diabetes receiving intensive insulin therapy. However, given the lack of significant post-hoc differences and the study’s limitations, the findings should be interpreted cautiously. Of the participants, 19.0% exhibited probable sarcopenia based on low handgrip strength.

**Supplementary Information:**

The online version contains supplementary material available at https://doi.org/10.1186/s13098-026-02258-1.

## Introduction

Diabetes is a chronic disease with multiple etiologies that results in hyperglycemia characterized by insulin resistance or deficiency [1]. The treatment strategy for diabetes varies according to the type, status of the disease, age, metabolic problems, accompanying complications and co-morbidities. Medical, nutrition and exercise therapies are effective in the treatment of the disease. However, diabetes education is essential for the effective implementation of these lifestyle changes [2].

Sarcopenia is a progressive and widespread skeletal muscle disorder characterized by low muscle strength, low muscle quantity or quality and low physical performance, which is associated with an increased likelihood of falls, fractures, physical disability and death [3]. In addition, sarcopenia is a condition generally seen with aging, but it has been reported that many chronic diseases, including diabetes, accelerate the development of sarcopenia [4]. Studies on the prevalence of sarcopenia in diabetes are limited and the prevalence of sarcopenia in diabetic patients has been found to be 15% [5, 6].

Intensive insulin therapy is a normal part of the treatment in type 1 diabetic patients. In type 2 diabetes, it is prescribed in conditions such as failure to respond to oral antidiabetic treatment, insulin deficiency symptoms, pregnancy, pancreatic dysfunction and corticosteroid use [7, 8].

Carbohydrate counting is a meal planning method that takes into account the effects of carbohydrate-containing foods on plasma glucose [9]. In addition, carbohydrate counting is considered the most effective method of calculating insulin doses associated with meals [10]. In the last decade, the increase in insulin analogues and insulin pump therapy has led to the widespread use of carbohydrate counting [11].

Carbohydrate counting provides more flexibility in the diet and in some cases can reduce the patient’s diabetes burden and improve quality of life [12, 13]. Carbohydrate counting is frequently recommended to diabetic patients as an alternative nutrition method to improve glycemic control and reduce the risk of developing acute and chronic complications [14]. However, the patient has to calculate the amount of carbohydrates taken in each meal, adjust bolus insulin doses by monitoring plasma glucose levels which may be difficult [15].

In a meta-analysis evaluating the effect of carbohydrate counting training on glycemic control and body weight in type 1 diabetes, it was reported that the method was effective in maintaining glycemic control in patients without exhibiting significant hypoglycemia and difference in body weight [16]. In another meta-analysis was reported that HbA1c levels decreased significantly in the carbohydrate counting training groups [12].

Although there is a lot of evidence on the effectiveness of carbohydrate counting training in type 1 diabetes, studies on its effect in type 2 diabetes is limited. In a study evaluating the effect of carbohydrate counting training in type 2 diabetic patients, a significant decrease in HbA1c levels was reported [17]. Another similar study in Denmark was reported that HbA1c levels decreased, in both groups however there was no significant difference between the two groups [18]. Another study evaluating the effect of carbohydrate counting training reported no significant difference in HbA1c and total insulin dose for both training and control groups [19].

The aim of this study was to evaluate the effects of advanced carbohydrate counting training on anthropometric/body composition, biochemical, and clinical parameters of patients with type 2 diabetes mellitus who receive intensive insulin therapy. In addition, this study also evaluated the sarcopenia status of the patients and the effect of carbohydrate counting training on sarcopenia.

## Method

### Study design and participants

This study was planned as a randomized, controlled, 24-week study and was conducted with the voluntary participation of individuals with type 2 diabetes who were treated with intensive insulin. The study sample consisted of patients who received outpatient treatment at the Uskudar Diabetes Polyclinic of the Fatih Sultan Mehmet Training and Research Hospital Additional Service Building of the Ministry of Health of the Republic of Turkey between August 2022 and December 2024.

Inclusion criteria included, a history of type 2 diabetes mellitus or using insulin for more than one year, being over 18 years old, receiving basal and bolus insulin treatment, not having received carbohydrate counting training before, not receiving treatment with sulfanylurea drugs from oral anti-diabetics. Exclusion criteria included being pregnant or breastfeeding or planning a pregnancy during the study period, discontinuing insulin treatment during the study period, having active proliferative retinopathy, severe nephropathy, severe or newly diagnosed cardiac disease within 6 months or stage III–IV heart failure, having dementia or psychosis and cognitive impairment, receiving glucocorticoid treatment, being fed enterally or parenterally and receiving nutrition treatment due to another/concomitant disease, having end-stage renal or hepatic/ renal disease (other than proteinuria); having liver disease/microangiopathy diseases, having clinical cancer, chronic respiratory disease or amputations, or having any disease or condition that would prevent completion of the study.

The study was conducted in accordance with the Declaration of Helsinki with the document number E-10840098-772.02-2030 and permission dated 24 March 2022 from the Istanbul Medipol University Non-Interventional Clinical Research Ethics Committee. Informed Voluntary Consent Form was obtained from all volunteers participating in the study. The trial is registered at ClinicalTrials.gov (registration no. NCT07016958).

### Sample size, randomization and formation of groups

A power analysis was conducted to determine the number of participants to be included in the study. In order to exceed the 80% power level, it was determined that the study should be conducted with 50 individuals (25 participants in the training and 25 participants in the control group), at a significance level was set at 5% with an effect size of 0.518 (df = 41; t = 1.683) [19]. Randomization was performed prior to participant enrollment by an independent statistician using a web-based random number generator with a 1:1 allocation ratio (simple randomization). No stratification factors were applied. Eligibility assessment was conducted by a physician who was unaware of the allocation sequence. Participants who met the inclusion criteria were enrolled sequentially according to their order of presentation. Group assignment was determined based on the predefined randomization list. The dietitian responsible for delivering the intervention accessed the allocation sequence at the time of enrollment to implement the assigned intervention. Due to the nature of the educational intervention, blinding was not feasible. Both participants and the researcher were aware of group allocation during the study. While the training group received advanced carbohydrate counting training in addition to standard medical and nutritional therapy, the control group received only the medical and nutritional therapy. Participants who dropped out during the study due to inclusion and exclusion criteria or refused to volunteer were not included in the data analysis at any time point in the study.

### Research protocol

All participants continued to receive routine medical and nutritional care at the diabetes center throughout the study. Physicians conducted follow-up visits every three months (approximately 15 min), during which laboratory tests were performed and antidiabetic medication regimens were adjusted based on clinical evaluation. Standard nutritional therapy was provided monthly by the institution’s dietitian (15–20 min per session). Anthropometric measurements were obtained and general dietary adherence was assessed. Standard nutritional therapy included individualized meal plans prepared according to estimated energy requirements. The participants’ personalized diet plans were created according to the basic nutritional principles for diabetes. Accordingly, in this diet plan, the proportion of energy from carbohydrates was 45–60%, from proteins 10–20%, and from fats 20–35%. Diet plans were structured based on meal timing and portion control principles and were reviewed monthly according to anthropometric measurements and dietary adherence. The routine diet plans did not include gram-based carbohydrate calculations or carbohydrate counting training. The institution’s dietitians were not informed about participants’ group allocation and continued routine care for both groups without modification. The research team did not interfere with or review routine dietary prescriptions in order to minimize performance bias. Carbohydrate counting was not implemented as part of routine care. In addition to routine care, the training group received structured carbohydrate counting training delivered individually by the research dietitian in three sessions. The control group did not receive additional training or study-related materials.

The training protocol, duration and content of each training session were decided by doctors and diabetes dietitians. All training sessions were delivered face-to-face and individually by the same research dietitian to ensure consistency. The dietitian holds a specialist dietitian qualification and certification in carbohydrate counting training. Individual training on carbohydrate counting were organized for patients in the training group in 3 sessions on different dates in parallel with carbohydrate counting training levels. The first level training session (90 min), provided basic information on nutrition and health and comprised of topics as, macronutrients in food groups and their effect on plasma glucose, glycemic index, healthy food preferences and meal planning, carbohydrate contents in various food items, concept of carbohydrate change (portions of foods containing 15 g of carbohydrate) were taught. A standardized slide-based curriculum was used for the first session to provide uniform delivery of fundamental carbohydrate counting principles. Subsequent sessions included structured practice on carbohydrate counting, insulin-to-carbohydrate ratio, and correction factor calculations using participants’ own dietary and glucose monitoring records. In the second session (45 min), the patients calculated the carbohydrate contents of the meals they consumed based on the knowledge from the previous session. In addition, the meal plans, snacking and main meal frequencies, physical activities, effect of insulin doses on plasma glucose, noted down by each patient in their data sheets of each patient were reviewed. In the third training (45 min), calculation of the total carbohydrate content of the consumed meal were repeated, in order to reinforce the patient’s training in this field. Furthermore, the patients were informed about the Insulin Correction Factor and Carbohydrate/Insulin ratios, and the insulin doses to be taken per meal were calculated and exemplified based on their meal plan data sheets. Four-day dietary and blood glucose monitoring forms were reviewed at second and third session. In cases of incomplete records, participants were asked to complete missing data before proceeding with subsequent training sessions. All records were checked by the research dietitian to maintain intervention fidelity and consistency across participants.

Participants were required to complete three training sessions. If a scheduled session was missed, participants were re-contacted and rescheduled. Follow-up outcome assessments were conducted only after completion of the training phase.

### Evaluation

A questionnaire form containing sociodemographic information was completed by face-to-face interview method. In addition, biochemical (HbA1c, fasting plasma glucose (FPG), C-peptide, total cholesterol, HDL, LDL, triglyceride, estimated glomerular filtration rate) and anthropometric and body composition (weight-height, BMI, fat mass, fat ratio, muscle mass, lean tissue mass, waist circumference), hand grip strength, blood pressure and physical activity measurements of the patients were taken at the beginning, week 12 and week 24 of the study.

Biochemical analyses of the participants were performed in the biochemistry laboratory of the hospital. The individuals participating in the study were asked to give blood sample in the morning after 10–12 h of fasting after dinner. Plasma glucose levels were analyzed by hexokinase method in colorimetric-spectrophotometric Aeroset (Roche Hitachi cobas 6000) autoanalyzer and total cholesterol, HDL and triglyceride levels were analyzed by enzymatic colorimetric method. LDL levels were calculated using the Fridewald equation [20]. In addition, HbA1c levels were measured by high-performance liquid chromatography (HPLC) method. Serum C-peptide levels were determined by Enzyme-Linked Immunosorbent Assay (ELISA) method (Roche Hitachi cobas 6000) using a commercial kit in an autoanalyzer.

Anthropometric measurements of the participants were taken by a dietician. The height of the participants was measured with a wall-type stadiometer with barefoot heels touching each other, arms hanging to the side, feet 45^o^ apart and head in the Frankfort plan. Body weight and body composition were measured with TANITA BC-418MA bioelectrical impedance analyzer. BMI values of the participants were calculated based on weight and height measurement data. In waist circumference measurement, the midpoint between the bottom of the rib bone and the iliac crest was determined and the circumference of this area was measured with a tape measure.

The participants’ hand grip strength was measured three times with a digital hand dynamometer (CAMRY-EH101, USA) and the mean value in kg was calculated. The validity and reliability of the hand dynamometer used for hand grip strength measurement was performed by Huang et al. [21]. According to the European Working Group on Sarcopenia in Older People 2 consensus, low muscle strength was defined as handgrip strength < 16 kg for women and < 27 kg for men [3]. Participants with handgrip strength below these thresholds were classified as having low muscle strength. In this context, individuals below this threshold value were evaluated as low muscle strength. Skeletal muscle mass data obtained from the BIA measurement results and skeletal muscle according to the height obtained from this data were calculated by dividing the skeletal muscle mass by the square of the height. In this context, in the European Working Group on Sarcopenia in Older People guideline, the threshold values for evaluation within the scope of sarcopenia were determined as 15 kg for women and 20 kg for men for skeletal muscle, while 5.5 kg/m^2^ for women and 7 kg/m^2^ for men for skeletal muscle by height [3]. In this context, individuals below this threshold value were evaluated as having low muscle mass.

In the Timed Up and Go test, the patient is asked to get up from the chair, walk at a normal walking speed on a flat surface of 3 m, turn around, walk back and sit back in the chair [22]. The time between the patient getting up from the chair, walking and sitting down again is recorded and the patient’s dynamic balance during walking is also evaluated. It has been reported that the Timed Up and Go test is a valid and reliable test in adults [23]. In addition, the threshold value of the test duration in the evaluation of sarcopenia was determined as 20 s in the European Working Group on Sarcopenia in Older People guideline and individuals above this threshold value were evaluated as low physical performance [3]. Sarcopenia measurements (handgrip strength, Timed Up and Go test) were performed under the supervision of a licensed physiotherapist within the scope of measurement technique standardization.

Blood pressure of the participants was measured with an automatic sphygmomanometer (SCIAN LD-533, Germany). Three repeated measurements were made on the right arm in the sitting position with the arm at the level of the heart and after a rest period of at least 10 min and the mean was calculated.

The International Physical Activity Questionnaire - Short Form (IPAQ-SF) was used to assess the physical activity of the participants. The IPAQ-SF consists of a total of 7 questions and the estimated duration of the participants’ mild, moderate and vigorous physical activity in the last 1 week is questioned. In addition, the daily sitting/resting times of the participants are also questioned. As a result of this information, the total weekly activity duration is obtained by multiplying the standard metabolic equivalent (MET) created according to the activity types by the number and duration of weekly activities. According to the MET value obtained, the physical activity status of the participants was classified as inactive (< 600 MET), minimally active (600–3000 MET) and active (> 3000 MET) [24].

In addition, a 24-hour retrospective food consumption record was applied to evaluate the energy and nutrient intakes. The calculation of the energy, macro and micro-nutrient intakes of the participants was carried out using the Turkish Nutrition Database Ebispro for Windows program (version 7.2) [25].

### Statistical analyses

The statistical analysis of the data obtained from the study was evaluated using the SPSS Statistics version 22.0 (IBM Corp., Armonk, NY, USA) package program. In order to evaluate the suitability of the data for normal distribution, One-Sample Kolmogorov-Smirnov test was conducted, and kurtosis and skewness of the data were evaluated. Two groups, the training group and the control group, were compared in this study. Between-group differences at each measurement point (baseline, 3rd month, and 6th month) were analyzed using the independent samples *t*-test for normally distributed data and the Mann–Whitney *U* test for non-normally distributed data. To assess within-group changes over time (baseline, 3rd month, and 6th month), the paired samples *t-*test and mixed-design ANOVA were employed for variables showing a normal distribution. When a significant main effect or interaction was detected in the ANOVA, pairwise comparisons were conducted using the Bonferroni correction to control for type I error. For variables that did not meet the assumption of normality, the Wilcoxon signed-rank test and Friedman test were used. All data have been provided with mean ± standard deviation (mean ± SD) and Median [IQR]. Categorical variables were compared using the Chi-square test, and when the expected cell count was less than 5, Fisher’s Exact Test was applied. These data were presented as frequency (n, %) values. The significance level between the data was accepted as *p* = 0.05. In multiple comparisons of measurements, Bonferroni correction was applied and *p* < 0.017 was taken. Effect size metrics were calculated to quantify the magnitude of significant findings. *η²* values were computed for parametric tests (Independent and Paired *t*-tests), *r* values for non-parametric tests (Mann–Whitney *U*, and Wilcoxon signed-rank tests), and partial *η²* values were obtained for mixed design ANOVA analyses. The effect size for the Friedman test was calculated using Kendall’s *W*. For chi-square tests, Cramer’s *V* and *φ* were used as the effect size measure. To control for baseline imbalance in C-peptide levels, an ANCOVA analysis was performed with 6-month HbA1c as the dependent variable, treatment group as the fixed factor, and baseline HbA1c and C-peptide levels as covariates. The graphical representations of the data were evaluated using the GraphPad Prism version 9.3.0 (GraphPad Software, San Diego, CA, USA) package program.

## Results

**Fig. 1:**
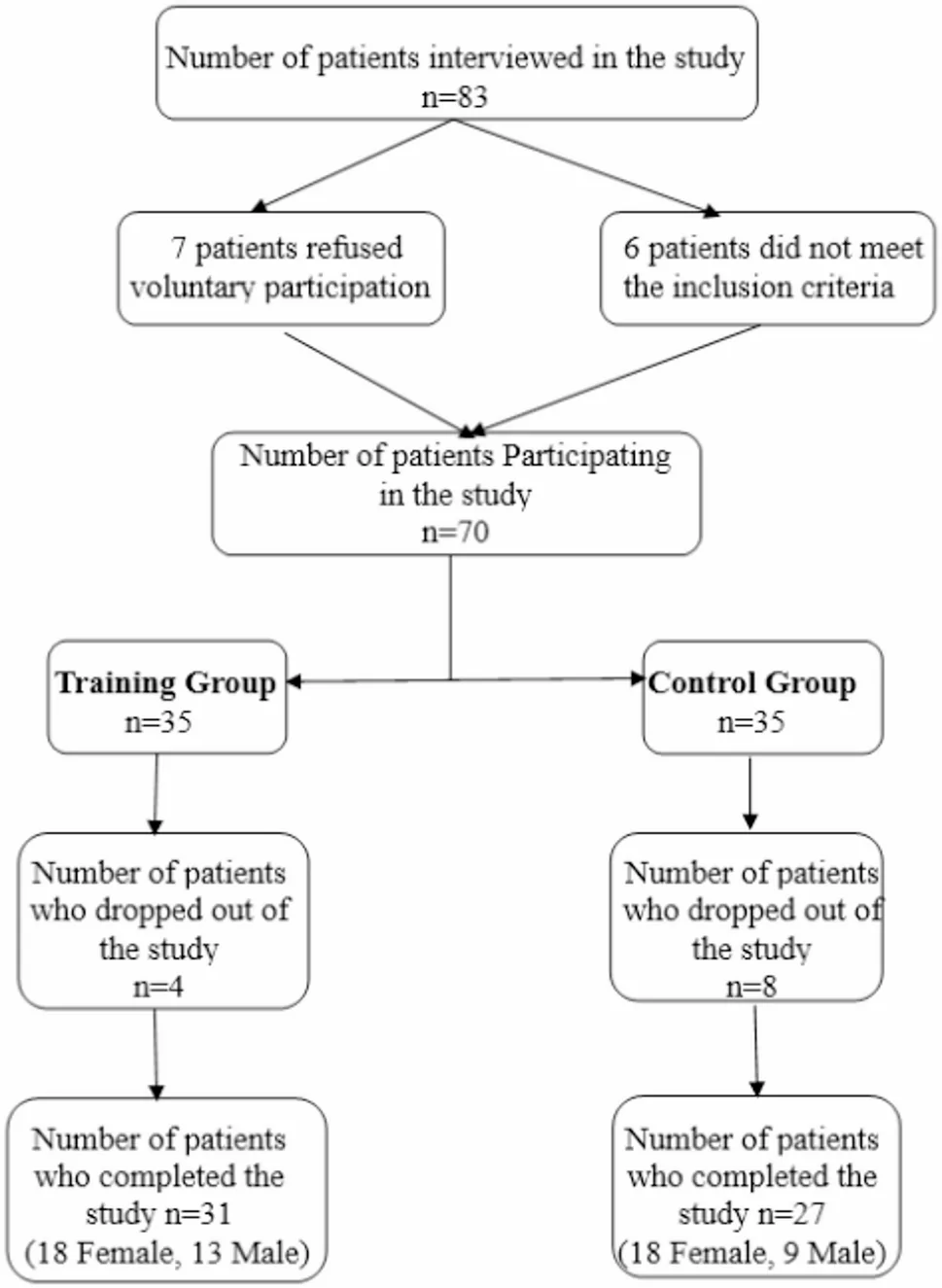
Workflow chart of the research plan

Eighty three patients were initially examined in the research center. The study started with a total of 70 patients and the patients were randomized into two groups (training and control) with 35 individuals in each. Five participants dropped out of the study because they discontinued intensive insulin treatment, 3 participants dropped out because they were not eligible for the study within the scope of the inclusion or exclusion criteria (diagnosed with cancer, liver diseases, etc.), and 4 participants dropped out because they later refused voluntary participation. Participants who completed both the 3-month and 6-month follow-up assessments were included in the final analysis. Participants who did not attend follow-up visits were considered drop-outs and were excluded from the per-protocol analysis. Thus, the study was completed with 31 patients in the training group and 27 patients in the control group (Fig. 1).

Baseline characteristics of participants who completed the study (*n* = 58) and those who dropped out (*n* = 12) were compared to evaluate potential attrition bias. No statistically significant differences were observed between the groups in terms of age (*p* = 0.582), sex distribution (*p* = 0.437), BMI (*p* = 0.230), baseline HbA1c levels (*p* = 0.418), or diabetes duration (*p* = 0.617) (Table 1).

**Table 1:** Baseline Characteristics of Participants According to Study Completion Status

Variable	Completed (*n* = 58)	Dropped-Out (*n* = 12)	*p*
Age (years)	59,81 ± 10,23	59,16 ± 9,77	0,582
Female n (%)	36 (62,1)	6 (50)	0,437
BMI (kg/m^2^)	30,13 ± 4,74	34,32 ± 13,50	0,230
HbA1c (%)	9,42 ± 1,73	9,08 ± 1,71	0,418
Diabetes Duration (years)	14,28 ± 10,49	11,25 ± 5,13	0,617

^a^ Chi-square, Mann Whitney *U*, Independent Samples *t*-test were applied to determine the significance of the data in this table

^b^ The level accepted for significance is *p* < 0.05

^c^ Mean *±* SD values has been provided for normally distributed (parametric) variables

There was no significant difference between the groups in terms of factors such as age, gender, duration of diabetes, basal insulin dose, bolus insulin dose and total insulin dose, education level, income level, using anti diabetic drug and comorbidities (Table 2). Of the participants, 41.9% (*n* = 13) in the training group and 11.1% (*n* = 3) in the control group had insufficient C-peptide levels. In addition, a significant difference was found between the groups in the C-peptide levels of the participants (*p* = 0.001). Given the baseline imbalance in C-peptide levels, an ANCOVA was performed with 6-month HbA1c as the dependent variable and baseline C-peptide and baseline HbA1c as covariates. Baseline C-peptide was not a significant predictor of 6-month HbA1c (*p* = 0.350, partial η²=0.016). Importantly, the group effect remained statistically significant after adjustment (*p* = 0.013, partial η²=0.109).

**Table 2:** Baseline characteristics of the participants

Variables	Training (*n* = 31)	Control (*n* = 27)	*p*	η²
Age (Year)	57.84 ± 9.04	62.07 ± 11.20	0.117	0.043
Diabetes Duration (Year)	15.0[5.5–23.0]	10.0[5.0–20.0]	0.349	0.049
Insulin Therapy Duration (Year)	4.0[1.0-12.5]	5.0[1.5–12]	0.950	0.125
Prandial Insulin (U/day)	18.0[13.5–24]	24.0[18.0–34.0]	0.069	0.001
Basal Insulin (U/day)	24.0[16.5–18.0]	26.0[21.0–30.0]	0.288	0.037
Total Insulin (U/day)	44.0[32.0–54.0]	46.0[40.0–64.0]	0.115	0.010
C-peptide (ng/mL)	1.26 ± 0.87	2.01 ± 0.81	0.001^c^	0.170
**C-peptide** ^**b**^	**n (%)**	**n (%)**	**p**	***η²***
Normal	18 (58.1)	24 (88.9)	0.009^c^	0.344
Inadequate	13 (41.9)	3 (11.1)		
**Income Level**				
Low	11 (35.5)	8 (29.6)	0.819	0.083
Middle	17 (54.8)	17 (63.0)		
High	3 (9.7)	2 (7.4)		
**Sex**				
Male	13 (41.9)	9 (33.3)	0.501	0.088
Female	18 (58.1)	18 (66.7)		
**Education Level**				
Primary Education	20 (64.5)	22 (81.5)	0.265	0.262
Secondary Education	6 (19.4)	2 (7.4)		
Undergraduate	4 (12.9)	1 (3.7)		
Postgraduate	1 (3.2)	2 (7.4)		
**Anti-diabetic Drug** ^**e**^				
Biguanide	6 (19.4)	2 (7.4)	0.070	0.257
Thiazolidinedione	1 (3.2)	-		
DPP-4 Inhibitor	6 (19.4)	3 (11.1)		
SGLT-2 Inhibitor	6 (19.4)	3 (11.1)		
**Comorbidities**				
Hypertension	20 (64.5)	20 (74.1)	0.433	0.103
Hyperlipidemia	13 (41.9)	6 (22.2)	0.111	0.210
Hypothyroidism	4 (12.9)	3 (11.1)	0.579	0.027

^a^ Chi-square, Mann Whitney *U*, Independent Samples *t*-test were applied to determine the significance of the data in this table. ^b^ In the study, the limit accepted as insufficiency in C-peptide level was accepted as 1.1 ng/mL. ^c^ The level accepted for significance is *p* < 0.05. ^d^ Mean *±* SD values has been provided for normally distributed (parametric) variables and Median [IQR] values for non-parametric variables ^e^ DPP-4: Dipeptidyl Peptidase 4 Inhibitor, SGLT-2: Sodium-Glucose Transport Protein 2 Inhibitor. ^f^ Effect sizes are presented as *η²*,* r*,* φ* or Cramer’s *V*, depending on the statistical test applied

The bolus insulin doses of the participants in the training group decreased by 7.48 ± 8.84 U/day in the week 12 and 7.64 ± 8.43 U/day in the week 24 compared to the beginning of the study (*p* = 0.034, η²=0.110). It was found that the frequency of hypoglycemia and hyperglycemia decreased significantly in the training group (*p* = 0.007, *η²*=0.161, *p* = 0.001, *η²*=0.249 respectively).

**Table 3:** Physiological and clinical parameters of participants over the weeks

Variable	Groups	Baseline	Week 12			Week 24			Total	
-	-	-	-	pd	η²		pe	η²	pf	η²
Daily Basal Insulin (U/day)	Training	24.0[16.5–28.0]	20.0[18.0–29.0]	0.819	0.030	22.0[18.0–31.0]	0.378	0.116	0.627	0.015
	Control	26.0[21.0–30.0]	24.0 [21.5–30]	0.781	0.036	27[20.0–35.0]	0.071	0.237	0.246	0.052
	p (*η²*)	0.288 (0.140)	0.370 (0.167)	0.999	0.000	0.151 (0.195)	0.059	0.247	0.297	0.021
Daily Prandial Insulin (U/day)	Training	18.0[13.5–24.0]	18.0[12.0–24.0]	0.902	0.016	14.0[12.0–24.0]	0.102	0.214	0.034^c^	0.110
	Control	24.0[18.0–34.0]	18.0[12.0-34.5]	0.204	0.167	18.0[12.0–30.0]	0.063	0.244	0.337	0.040
	p (*η²*)	0.069 (0.239)	0.224 (0.050)	0.320	0.131	0.173 (0.111)	0.013^g^	0.325	0.024^c^	0.064
Total Insulin (U/day)	Training	44.0[32.0–54.0]	38.0[31.0-53.5]	0.726	0.045	38.0 [29.5–52.5]	0.486	0.091	0.292	0.040
	Control	46.0[40.0–64.0]	46.0[35.5–61.5]	0.306	0.134	48.0 [33.5–63.5]	0.509	0.087	0.674	0.015
	p (*η²*)	0.079 (0.230)	0.248 (0.127)	0.623	0.064	0.131 (0.172)	0.318	0.131	0.316	0.020
Hypoglycemia (Month)	Training	1.0[0.0–2.0]	0.0[0.0–1.0]	0.101	0.215	0.0 [0.0–0.0]	0.004^g^	0.381	0.007^c^	0.161
	Control	0.0[0.0–2.0]	0.0[0.0–1.0]	0.643	0.061	0.0 [0.0–1.0]	0.958	0.001	0.816	0.008
	p (*η²*)	0.306 (0.118)	0.626 (0.066)	0.081	0.229	0.062 (0.271)	0.024	0.295	0.076	0.045
Hyperglycemia (Month)	Training	3.0[1.0–10.0]	1.0[0.0-4.5]	0.030	0.285	1.0[0.0-2.5]	0.001^g^	0.461	0.001^c^	0.249
	Control	4.0[1.0-6.5]	3.0[2.0–4.0]	0.384	0.114	3.0[2.0-4.5]	0.245	0.152	0.726	0.012
	p (*η²*)	0.357 (0.066)	0.767 (0.179)	0.021	0.303	0.410 (0.302)	0.001^g^	0.477	0.004^c^	0.096
Body Weight (kg)	Training	81.10 ± 14.11	80.08 ± 14.70	0.108	0.084	79.23 ± 14.41	0.010^g^	0.200	0.024^c^	0.120
	Control	77.67 ± 12.04	78.88 ± 12.81	0.142	0.080	77.66 ± 11.66	0.991	0.465	0.482	0.027
	p (*η²*)	0.329 (0.017)	0.743 (0.002)	0.972	0.149	0.654 (0.004)	0.160	0.036	0.084	0.013
BMI (kg/m^2^)	Training	29.56 ± 4.91	29.22 ± 4.91	0.271	0.040	28.78 ± 4.91	0.030	0.148	0.011^c^	0.146
	Control	30.79 ± 4.56	31.25 ± 4.94	0.139	0.082	30.56 ± 4.12	0.704	0.005	0.767	0.010
	p (*η²*)	0.329 (0.017)	0.122 (0.042)	0.872	0.001	0.145 (0.038)	0.121	0.005	0.177	0.027
Waist Circumference (cm)	Training	94.51 ± 12.18	93.58 ± 12.45	0.202	0.029	93.14 ± 11.58	0.077	0.059	0.032^c^	0.111
	Control	100.00 ± 8.94	100.19 ± 8.61	0.196	0.002	97.62 ± 11.31	0.491	0.061	0.584	0.020
	p (*η²*)	0.059 (0.062)	0.021^c^ (0.087)	0.888	0.007	0.148 (0.037)	0.097	0.064	0.317	0.025
Fat Weight (kg)	Training	24.4[17.5–30.6]	23.6[16.8–31.5]	0.676	0.054	22.1[17.5–29.7]	0.716	0.047	0.525	0.021
	Control	24.9[19.8–28.2]	24.9[21.5–31.6]	0.882	0.019	26.5[19.8–29.3]	0.629	0.063	0.740	0.011
	p (*η²*)	0.682 (0.096)	0.557 (0.083)	0.848	0.025	0.621 (0.102)	0.955	0.007	0.825	0.005
Fat (%)	Training	30.67 ± 8.53	30.96 ± 9.05	0.661	0.008	31.11 ± 8.78	0.692	0.014	0.880	0.004
	Control	33.4 ± 7.14	33.11 ± 7.46	0.374	0.020	33.67 ± 7.56	0.788	0.007	0.386	0.035
	p (*η²*)	0.190 (0.030)	0.334 (0.017)	0.866	0.017	0.243 (0.024)	0.639	0.011	0.826	0.003
Muscle Weight (kg)	Training	42.4[38.5–57.0]	42.6[37.25–56.9]	0.259	0.148	46.8[37.9–56.2]	0.667	0.192	0.032^c^	0.111
	Control	43.2[35.6–47.2]	44.7[38.6–51.2]	0.056	0.251	43.0[38.4–49.5]	0.462	0.154	0.282	0.047
	p (*η²*)	0.135 (0.168)	0.428 (0.056)	0.557	0.077	0.344 (0.105)	0.674	0.007	0.099	0.001
Fat-Free Mass (kg)	Training	55.7[47.9–61.8]	55.1[46.65–60.8]	0.061	0.245	52.7[46.7–60.2]	0.001^g^	0.449	0.001^c^	0.244
	Control	49.2[44.9–59.3]	49.1[45.5–60.2]	0.493	0.090	50.1[42.9–58.6]	0.539	0.080	0.825	0.007
	p (*η²*)	0.152 (0.183)	0.298 (0.117)	0.365	0.118	0.260 (0.149)	0.009^g^	0.341	0.225	0.067
HbA1c (%)	Training	9.66 ± 1.83	8.99 ± 1.36	0.007^g^	0.216	8.55 ± 1.37	0.001^g^	0.294	0.006^c^	0.181
	Control	9.14 ± 1.60	9.10 ± 1.75	0.911	0.001	9.26 ± 1.73	0.735	0.005	0.901	0.067
	p (*η²*)	0.257 (0.023)	0.798 (0.001)	0.079	0.059	0.088 (0.051)	0.032	0.093	0.040^bc^	0.068
HDL Cholesterol (mg/dL)	Training	44.6[40.9–63.4]	49.1[40.6–61.9]	0.567	0.075	50.2[41.1–60.7]	0.239	0.154	0.744	0.010
	Control	47.0[37.0–56.0]	44.6[39.0–55.0]	0.486	0.091	48.4[39.9–55.0]	0.904	0.016	0.712	0.013
	p (*η²*)	0.459 (0.097)	0.239 (0.154)	0.993	0.001	0.238 (0.154)	0.353	0.121	0.650	0.008
LDL Cholesterol (mg/dL)	Training	107.74 ± 49.44	115.57 ± 33.81	0.111	0.048	113.89 ± 38.67	0.269	0.022	0.409	0.031
	Control	125.83 ± 56.70	103.48 ± 38.67	0.313	0.081	105.31 ± 30.36	0.159	0.072	0.478	0.027
	p (*η²*)	0.282 (0.021)	0.214 (0.028)	0.633	0.011	0.362 (0.015)	0.849	0.015	0.698	0.006
Total Cholesterol (mg/dL)	Training	175.24 ± 60.77	197.14 ± 41.71	0.031	0.145	204.71 ± 41.94	0.015^g^	0.177	0.048^c^	0.109
	Control	200.32 ± 45.03	186.08 ± 41.75	0.055	0.135	183.66 ± 45.71	0.158	0.124	0.682	0.015
	p (*η²*)	0.083 (0.053)	0.325 (0.017)	0.441	0.011	0.028^c^ (0.062)	0.426	0.009	0.432	0.016
TG (mg/dL)	Training	131.9[85.2-173.3]	112.2[79.3-192.3]	0.374	0.116	128.1[77.8-190.8]	0.638	0.062	0.448	0.029
	Control	175.4[116.8–217.0]	183.4[136.3–232.0]	0.801	0.033	176.8[134.7-211.4]	0.949	0.008	0.919	0.168
	p (*η²*)	0.027^c^ (0.290)	0.030^c^ (0.284)	0.541	0.080	0.114 (0.248)	0.786	0.035	0.500	0.013
GFR (ml/min/ 1.73 m^2^)	Training	94.3[76.0-104.0]	92.9[78.9-105.5]	0.922	0.012	94.1[78.1-104.3]	0.779	0.036	0.965	0.001
	Control	83.3[69.6–95.2]	85.5[71.8–95.2]	0.407	0.108	84.5 [65.8–96.3]	0.790	0.035	0.691	0.014
	p (*η²*)	0.160 (0.168)	0.119 (0.203)	0.636	0.062	0.209 (0.146)	0.970	0.005	0.832	0.003
FPG (mg/dL)	Training	191.95 ± 82.06	176.53 ± 73.01	0.191	0.035	167.47 ± 69.15	0.098	0.101	0.832	0.054
	Control	175.09 ± 75.97	173.92 ± 67.20	0.949	0.443	149.51 ± 78.00	0.200	0.071	0.199	0.007
	p (*η²*)	0.423 (0.012)	0.400 (0.000)	0.390	0.010	0.361 (0.015)	0.043	0.103	0.230	0.026
Systolic Blood Pressure (mmHg)	Training	144.77 ± 21.93	135.35 ± 19.12	0.014^g^	0.034	139.41 ± 18.76	0.704	0.204	0.428	0.031
	Control	132.89 ± 28.59	140.04 ± 21.51	0.493	0.028	141.08 ± 14.17	0.297	0.028	0.675	0.018
	p (*η²*)	0.151 (0.047)	0.473 (0.011)	0.209	0.005	0.637 (0.002)	0.604	0.007	0.457	0.019
Diastolic Blood Pressure (mmHg)	Training	79.32 ± 14.50	74.35 ± 10.06	0.040	0.123	79.51 ± 12.12	0.423	0.004	0.031^c^	0.129
	Control	78.54 ± 12.58	77.4 ± 10.61	0.545	0.012	83.58 ± 11.09	0.235	0.046	0.188	0.057
	p (*η²*)	0.847 (0.000)	0.176 (0.025)	0.056	0.061	0.345 (0.025)	0.162	0.023	0.007^c^	0.090

^a^ For the significance of the data in this table, Mann Whitney *U*, Independent Samples *t-*test, Wilcoxon, Dependent Samples *t-*test and Friedman test were applied. ^b^Mixed Design ANOVA method was used in this data analysis.^c^ The accepted level for significance is *p* < 0.05. ^d^ Significance analysis of paired assessment of baseline and week 12 measurements ^e^Significance analysis of paired assessment of baseline and week 24 measurements ^f^Significance analysis of triple assessment of baseline, week 12 and week 24 measurements ^g^ Bonferroni correction was applied for Post Hoc Analyses: *p* < 0.017. ^h^ Mean *±* SD values has been provided for normally distributed (parametric) variables and Median [IQR] values for non-parametric variables. ^i^ BMI: Body Mass Index, HbA1c: Hemoglobin A1c, HDL: High Density Lipoprotein, LDL: Low Density Lipoprotein, GFR: Glomerular Filtration Rate, FPG: Fasting Plasma Glucose. ^j^Effect sizes are presented as *η²*,* r*, partial *η²*, or Kendall’s *W* depending on the statistical test applied

**Fig. 2:**
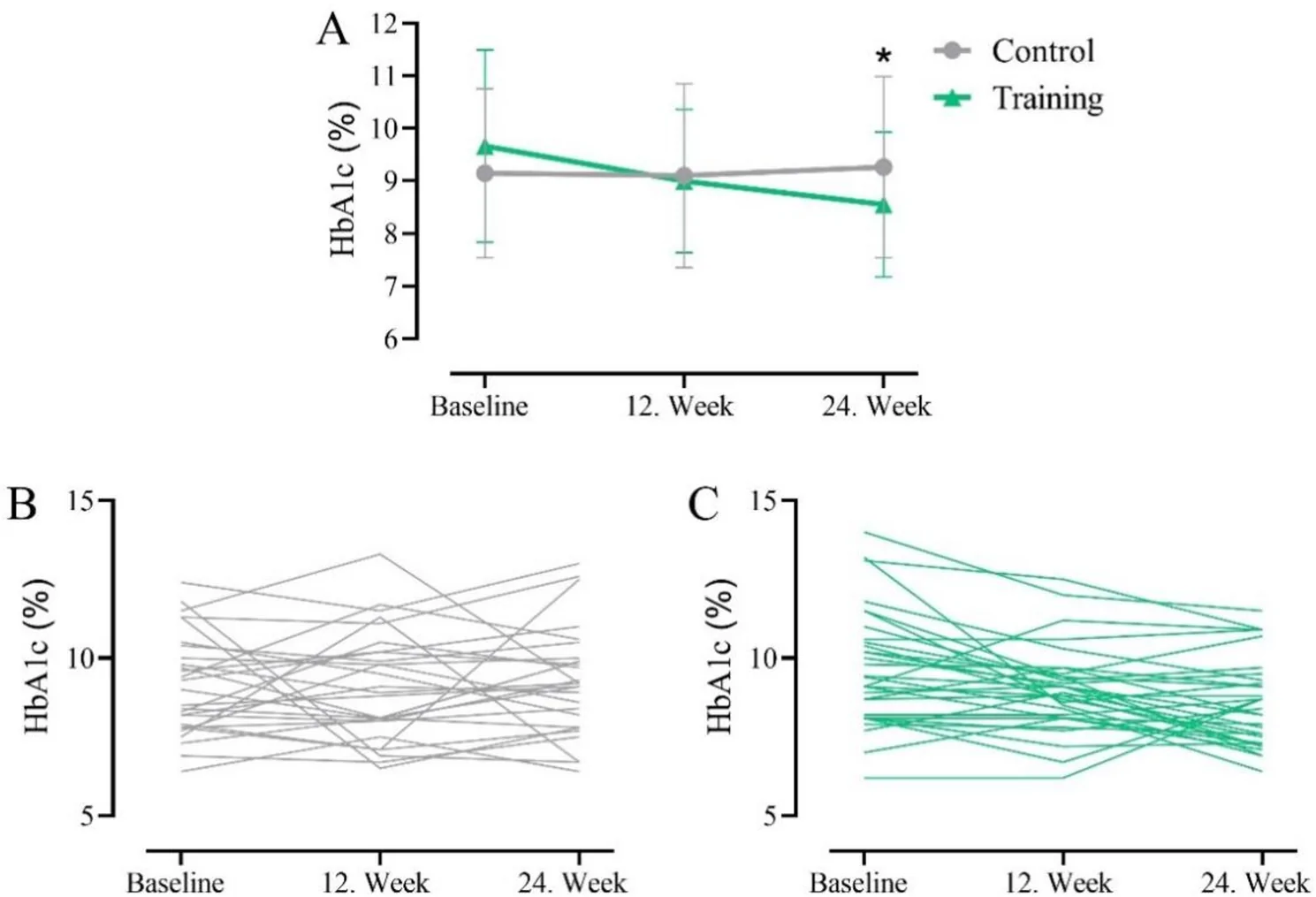
Graphic representation of HbA1c changes over the weeks. **A**. Mean (± SD) HbA1c values at baseline, week 12, and week 24 in the training and control groups. The asterisk (*) indicates a statistically significant time-by-group interaction identified by the mixed-design ANOVA (*p* = 0.040). However, Bonferroni-adjusted post-hoc comparisons were not statistically significant. **B**. Spaghetti plot of individual HbA1c trajectories in the control group. **C.** Spaghetti plot of individual HbA1c trajectories in the training group

There was a decrease of 1.04 ± 1.01 in HbA1c values of patients in the training group week 12 and 1.56 ± 1.35 week 24 compared to the baseline (*p* = 0.007, *η²=*0.216, *p* = 0.001, *η²=*0.294 respectively). A statistically significant difference was found the training group in the change in HbA1c levels over time as compared to the beginning (*p* = 0.006, *η²=*0.181). A statistically significant difference was found between the groups in the change in HbA1c levels over time as compared to the beginning (*p* = 0.040, *η²*=0.068). However, Bonferroni-adjusted pairwise comparisons showed no significant differences between specific time points (*p* = 0.778). The changes in the participants’ HbA1c levels are shown in Fig. 2; Table 3. Although a significant time-by-group interaction was observed (mixed-design ANOVA, *p* = 0.040), Bonferroni-adjusted post-hoc comparisons were not statistically significant.

In the week 12 measurements of the training group, body weight decreased by 2.53 ± 2.47 kg compared to the baseline values, the changes were not statistically significant. On the week 24 however, mean body weight in the training group decreased by 2.98 ± 2.97 kg compared to the initial measurements and the decrease was statistically significant (*p* = 0.024, *η²=*0.120). In addition, when the beginning, week 12, and week 24 were evaluated together, a significant overall time effect was observed for muscle weight in the training group (*p* = 0.032, *η²*=0.111). However, pairwise comparisons were not significant. In contrast, fat-free mass values in the training group significantly decreased at week 24 compared to baseline (*p* = 0.001, *η²*=0.449). When the time-dependent changes in the fat-free mass data of the participants in the training group were examined at all measurement points, a significant difference was detected (*p* = 0.001, *η²*=0.244). (Table 3).

Significant differences between the groups were observed for triglyceride levels at baseline (*p* = 0.027, *η*² *=*0.290) and week 12 (*p* = 0.030, *η*² *=*0.284), and for total cholesterol at week 24 (*p* = 0.028, *η*² *=*0.062). In addition, within the training group, total cholesterol levels significantly increased compared to baseline at week 24 (mean increase: 40.45 ± 56.48 mg/dL, *p* = 0.015, *η*² *=* 0.177). The overall effect over time was also significant (*p* = 0.048, *η*² = 0.109) (Table 3).

Bioimpedance, handgrip strength and Timed Up and Go test results of the participants were included in the study and the individuals were evaluated as “low” and “normal” based on the cut-off points determined by the European Working Group on Sarcopenia in Older People and Christopher et al. [3, 23]. There was no significant difference between the groups in baseline handgrip strength, mean value being 24.7 ± 9.90 kg. However, the proportion of patients with low muscle strength in the hand grip strength test was 12.9% (*n* = 4) in the training group, 25.9% (*n* = 7) in the control group and 19.0% in the whole sample (control and training). (Table 4). Baseline median muscle mass and mean muscle mass according to height of both groups were found to be 42.8 [36.8–54.5] kg and 17.23 ± 2.99 kg/m^2^ respectively. However, a significant overall time effect was observed for skeletal muscle mass in the training group (*p* = 0.032, *η*² = 0.111), although pairwise comparisons between time points were not statistically significant.

A significant difference was also found between the groups in the week 12 and 24 measurements of Timed Up and Go test in participants. The baseline means of the Timed Up and Go test in both groups was 8.10 ± 2.29 s. There was no significant difference between the groups regarding time-dependent changes in the parameters. In addition, no statistically significant change was found in the time-dependent changes of baseline, week 12 and week 24 IPAQ- SF scores and between the groups.

**Table 4:** Comparison of sarcopenia related findings in participants

Variable	Groups	Baseline			Week 12			Week 24						
		-		-	*p* ^d^		η²		*p* ^e^	η²		*p* ^f^		η²
Hand Grip Strength (kg)	Training	23.3[18.2–31.7]		23.8[18.5–32.9]	0.777		0.137	23.8[19.1–33.0]	0.737	0.152		0.933		0.039
	Control	19.6[17.5–30.5]		20.2[16.1–29.8]	0.583		0.069	20.9[15.8–30.4]	0.654	0.027		0.674		0.009
	p (*η²*)	0.290 (0.131)		0.177 (0.182)	0.727		0.142	0.435 (0.155)	0.585	0.070		0.666^b^		0.011
Skeletal Muscle Mass (kg)	Training	42.4[38.5–57.0]		42.6[37.25–56.9]	0.259		0.148	46.8[37.9–56.2]	0.667	0.192		0.032^c^		0.111
	Control	43.2[35.6–47.2]		44.7[38.6–51.2]	0.056		0.251	43.0[38.4–49.5]	0.462	0.154		0.282		0.047
	p (*η²*)	0.135 (0.167)		0.428 (0.056)	0.557		0.077	0.344 (0.105)	0.674	0.007		0.099		0.001
Skeletal Muscle Mass by Height (kg/m^2^)	Training	17.35 ± 3.06		17.25 ± 3.21	0.582		0.010	17.21 ± 3.25	0.664	0.006		0.803		0.111
	Control	17.09 ± 2.97		17.91 ± 3.03	0.034		0.162	17.68 ± 3.23	0.354	0.033		0.108		0.047
	p (*η²*)	0.729 (0.002)		0.429 (0.011)	0.122		0.041	0.584 (0.005)	0.570	0.006		0.072^b^		0.023
Timed Up and Go test (sec.)	Training	7.65 ± 2.44		7.47 ± 2.54	0.483		0.017	7.45 ± 2.23	0.555	0.012		0.967		0.001
	Control	8.61 ± 2.01		8.48 ± 1.98	0.582		0.012	8.67 ± 2.27	0.789	0.003		0.253		0.051
	p (*η²*)	0.050 (0.044)		0.033^c^ (0.048)	0.366		0.014	0.020^c^ (0.070)	0.703	0.003		0.635		0.008
IPAQ-SF scores (MET)	Training	5040.0[2229.3-6387.5]		3652[2848.8-4841.2]	0.552		0.078	3606.0[3102.0-5351.0]	0.649	0.059		0.436		0.027
	Control	4410.0[2684.7–5077.0]		4058.0[3156.0-4590.0]	0.585		0.071	3900.0[2827.5-4578.5]	0.773	0.037		0.991		0.019
	p (*η²*)	0.629 (0.063)		0.882 (0.019)	0.384		0.114	0.749 (0.042)	0.887	0.018		0.687		0.006
	**Training**							**Control**						
	**Baseline**	**Week 12**	**Week 24**			**Baseline**		**Week 12**			**Week 24**			
	**n (%)**	**n (%)**	**n (%)**			**n (%)**		**n (%)**			**n (%)**		**p**	***η²***
Hand Grip Strength														
Normal	27 (87.1)	26 (83.9)	26 (83.9)			20 (74.1)		19 (70.4)			17 (63.0)		0.223	0.026
Low muscle strength	4 (12.9)	5 (16.1)	5 (16.1)			7 (25.9)		8 (29.6)			10 (37.0)			
Skeletal Muscle Mass														
Normal	31 (100)	31 (100)	31 (100)			27 (100)		27 (100)			27 (100)		-	-
Low muscle mass	-	-	-			-		-			-			
Skeletal Muscle Mass by Height														
Normal	31 (100)	31 (100)	31 (100)			27 (100)		27 (100)			27 (100)		-	-
Low muscle mass	-	-	-			-		-			-			
Time Up and Go Tests														
Normal	31 (100)	31 (100)	31 (100)			27 (100)		27 (100)			27 (100)		-	-
Low physical performance	-	-	-			-		-			-			
Subjective Assessment														
Slow and changing tempo	3(9.7)	2 (6.5)	1 (3.2)			6 (22.2)		5 (18.5)			4 (14.8)		0.135	0.034
Short stride	4 (14.8)	2 (6.5)	1 (3.2)			4 (14.8)		4 (14.8)			3 (11.1)		0.135	0.025
Holding on to the wall	-	-	-			-		1 (3.7)			-		0.368	0.017
Mould	1(3.2)	-	-			-		1 (3.7)			1 (3.7)		1.000	0.000
Loss of balance	-	-	-			1 (3.7)		-			-		0.368	0.017
Short arm swing	1 (3.2)	2 (6.5)	-			4 (14.8)		4 (14.8)			3 (11.1)		0.223	0.026
Shuffle	1 (3.2)	-	-			2 (7.4)		1 (3.7)			1 (3.7)		0.135	0.034
Inability to use walking aids properly	-	-	-			-		-			-		-	-
IPAQ-SF Scores														
Sedentary	-	-	-			-		-			-		0.069	0.046
Minimal Active	13 (41.9)	9 (29.0)	7 (22.6)			10 (37.0)		5 (18.5)			9 (33.3)			
Active	18 (58.1)	22 (71.0)	24 (77.4)			17 (63.0)		22 (81.5)			18 (66.7)			

^a^ Chi-square, Mann Whitney *U*, Independent Samples *t*-test, Wilcoxon, Dependent Samples *t*-test and Friedman test were applied to the significance of the data in this table. ^b^ Mixed Design ANOVA method was used in this data analysis. ^c^ The level accepted for significance is *p* < 0.05. ^d^ Significance analysis of paired assessment of baseline and week 12 measurements ^e^ Significance analysis of paired assessment of baseline and week 24 measurements ^f^ Significance analysis of triple assessment of baseline, week 12 and week 24 measurements. ^g^ Bonferroni correction was applied for Post Hoc Analyses: *p* < 0.017. ^h^ Mean ± SD values has been provided for normally distributed (parametric) variables and Median [IQR] values for non-parametric variables. ^i^ The cut-off points accepted as inadequacy in the study were accepted as 16 kg for women and 27 kg for men for hand grip strength, 15 kg for women and 20 kg for men for skeletal muscle mass, 5.5 kg/m^2^ for women and 7 kg/m^2^ for men for skeletal muscle mass according to height, and 20 s for the Timed Up and Go test. ^j^ Effect sizes are presented as *η²*, *r*, partial *η²*, or Kendall’s *W* depending on the statistical test applied

A significant difference was found between the groups regarding protein intake at baseline, and in the energy, carbohydrate, protein, fat, saturated fatty acids, and cholesterol intake on week 12. On evaluating week 12 measurements as per baseline values, it was determined that the ratio of energy coming from protein in the whole sample increased which were statistically significant (*p* = 0.001, *η²=*0.299). When the week 24 values were evaluated according to the baseline values, a significant decrease in dietary fiber intake was observed in the training group (*p* = 0.007, η²=0.270). In addition, a significant change in dietary fiber intake over the three assessment time points was observed in the training group (*p* = 0.009, η²=0.174). In addition, the decrease in monounsaturated fatty acids in the whole sample was also found to be statistically significant (*p* = 0.048, *η²=*0.062) (Table S1).

## Discussion

This is one of the first studies aiming to examine sarcopenia in a type 2 diabetic patient group receiving intensive insulin therapy, where glycemic control is difficult, and evaluate the effect of advanced carbohydrate counting training on sarcopenia. Few studies have been conducted evaluating the effect of advanced carbohydrate counting on diabetic parameters of type 2 diabetic patients and this is the first one in Turkish population with type-2 diabetes. In this study, diabetic patients who received standard medical and nutrition therapy were randomized and divided into 2 groups: the control group and the advanced carbohydrate counting training group. It was determined that there was a significant decrease in hypoglycemia frequency, hyperglycemia frequency and bolus insulin doses in participants who received advanced carbohydrate counting training compared to the control group.

Carbohydrate counting training is designed for diabetic patients and includes meal planning strategies for controlling the effects of carbohydrates on plasma glucose [26]. In four meta-analysis studies, evaluating the effectiveness of carbohydrate counting training in type 1 diabetic patients, a statistically significant decrease in HbA1c levels was found in the group that received advanced carbohydrate counting training compared to the control group [12, 16, 27, 28].

Studies on the effect of advanced carbohydrate counting training in type 2 diabetes are limited, but in a study conducted in which the effect of this training was evaluated in this patient group, a significant decrease in HbA1c levels was found in the training group [17]. In another study evaluating the effectiveness of advanced carbohydrate counting training in a similar patient group, it was found that there was a decrease in HbA1c levels, but this decrease was not statistically significant. In addition, similar to the findings of this study, no significant difference was observed in total insulin dose in both groups [19].

In another study by Ewers et al., evaluating the effect of basic carbohydrate counting training in type 2 diabetic patients, it was reported that there was no significant decrease in HbA1c levels in the training group [18]. The decrease in HbA1c levels observed in this study was consistent with previous literature findings. Although a significant time-by-group interaction was detected, post-hoc comparisons did not remain significant after Bonferroni correction. Therefore, these findings should be considered preliminary and interpreted with caution and should not be regarded as definitive evidence of a superior intervention effect. The within-group reduction in HbA1c observed in the training group may indicate a potential benefit of advanced carbohydrate counting training in individuals with type 2 diabetes; however, this observation requires confirmation in larger, adequately powered randomized controlled trials.

In addition, significant weight loss was detected in the individuals in the training group at the end of the study (after 24 weeks) despite no significant change in physical activity levels. The improvement in HbA1c without a significant difference in physical activity levels is an important finding for the effectiveness of carbohydrate counting training. The weight loss in the patients in the training group may also have an effect on the improvements in HbA1c and other diabetic parameters of the patients. Although a baseline imbalance in C-peptide levels was observed between groups (*p* = 0.001, η²=0.170), ANCOVA analysis demonstrated that baseline C-peptide was not an independent predictor of 6-month HbA1c (*p* = 0.350, partial η²=0.016). However, after adjusting for ANCOVA, the effect of the training group on HbA1c levels was found to be statistically significant (*p* = 0.013, partial η²=0.109). These findings suggest that the observed improvement in HbA1c cannot be explained solely by baseline differences in endogenous insulin secretion.

In two meta-analysis studies, evaluating advanced carbohydrate counting training in type 1 diabetes, contrary to the findings in this study, it was reported that there was no significant difference in the frequency of hypoglycemia [16, 29]. In addition, in the study conducted on type 2 diabetes patients, it was reported that the frequency of hypoglycemia decreased in a similar direction to this study, but this change was not statistically significant [17]. It is thought that the statistically significant decrease in the frequency of hypoglycemia detected in the training group in this study is related to the decrease in bolus insulin doses.

Many studies examining the effects of advanced carbohydrate counting training have focused only on glycemic control parameters. Therefore, studies on the effects of this training on other blood parameters are limited. However, similar to this study, a few studies have found no significant difference in HDL-C and LDL-C levels [18, 30]. Furthermore, this study found no significant difference in GFR and FPG levels, but a statistically significant increase in total cholesterol levels was found in the training group. In addition, there was no statistically significant difference in energy, total fat and cholesterol intake over time compared to baseline measurements in the training group participants. Although there was no significant difference in HDL-C and LDL-C levels among the participants, this isolated increase in total cholesterol levels may have limited clinical significance. In this context, the increase in total cholesterol levels, independent of cholesterol intake, should be interpreted with caution. Indeed, it is thought that this finding may reflect the biological variability of the participants rather than the effect of the intervention.

In the meta-analysis conducted which evaluated the effect of advanced carbohydrate counting training in type 1 diabetes, it was determined that there was no significant difference in the body weight of the patients in the training group [16]. In another similar meta-analysis was reported that there was a decrease in the BMI values of the patients, but this change was not statistically significant [27]. In two other studies conducted on type 2 diabetes patients, it was reported that there was no significant change in body weight [18, 19]. In this study, although medical and nutritional therapy other than carbohydrate counting training were similar in both groups, a significant decrease was observed in body weight and BMI in the training group. There are studies that observed significant weight loss in patients given carbohydrate counting training, similar to this study [31–33]. The weight loss observed after carbohydrate counting training may have developed due to reasons such as improvement in nutritional status and increased physical activity with awareness achieved with the training [31]. In fact, it was determined that there was a non-significant decrease in the energy intake of the participants in the training group in week 12 and week 24, as compared to the baseline. The decrease in energy intake that occurred in the training group is supported by the study conducted by Bayram et al., who evaluated the effect of carbohydrate counting training in patients with type 1 diabetes and reported similar findings [30].

Sarcopenia is associated with increased hospitalization rates, cardiovascular complications, and mortality in diabetes [4, 34]. In addition, insulin resistance and oxidative stress play a key role in the pathophysiology of sarcopenia [34]. Therefore, it is reported that the prevalence of sarcopenia in diabetic patients is 2–3 times higher than in healthy individuals [35]. In a study examining the prevalence of sarcopenia in type 2 diabetic patients, hand grip strength of the participants was evaluated, and the prevalence of sarcopenia was found to be 16% [34]. In a meta-analysis evaluating 45 studies on the prevalence of sarcopenia in diabetes, muscle mass was analyzed with methods such as BIA and DEXA and the prevalence of sarcopenia in diabetes was reported to be 18% [36]. Another meta-analysis of epidemiological studies on the prevalence of sarcopenia in patients with type 2 diabetes, reported that the prevalence of sarcopenia in different populations varied between 7 and 29.3% [37].

In the present study, the median skeletal muscle mass of the participants was above the EWGSOP2 cut-off values, and no participant was classified as having low muscle mass and low physical performance. Accordingly, none of the participants fulfilled the EWGSOP2 diagnostic criteria for confirmed sarcopenia. However, 19.0% of the participants exhibited low muscle strength based on handgrip assessment and were therefore classified as having probable sarcopenia according to the EWGSOP2 consensus [3]. In this study, no participants were found who could be described as having low muscle mass. From a physiological perspective, a progressive and generalized loss of muscle mass can be observed after the age of 40. It is estimated that the decrease in muscle mass is 8% per decade until the age of 70 and 15–25% per decade after the age of 70 [38]. Although the participants in the study were in an advanced stage of the disease, the fact that they did not have sarcopenia in terms of muscle mass is thought to be related to the average age and relatively high BMI values of the participants included in the study. Indeed, increasing age and decreasing BMI values in individuals with type 2 diabetes are associated with a significant increase in the prevalence of sarcopenia [37].

A significant decrease in weight was detected in the individuals in the training group at the end of 24 weeks. This may be related to the awareness that the participants had developed only towards the carbohydrate content of foods. However, due to the high risk of sarcopenia in this patient group and the importance of muscle mass and function in the prognosis of the disease, it is important to create nutritional programs for patients by taking these factors into consideration and to increase the patient’s compliance with the nutritional program in order to prevent the development of sarcopenia in later stages of life.

In conclusion, advanced carbohydrate counting training was associated with reductions in bolus insulin dose and hypoglycemia frequency in the intervention group. Although a significant time-by-group interaction was observed for HbA1c, post-hoc comparisons after Bonferroni correction were not statistically significant. Therefore, the findings regarding HbA1c should be interpreted with caution and should not be considered definitive evidence of superior glycemic improvement. Carbohydrate counting training may represent a supportive strategy for glycemic management in individuals with type 2 diabetes receiving intensive insulin therapy; however, larger, adequately powered studies with longer follow-up are needed to confirm its clinical effectiveness and long-term impact.

This study has several methodological limitations. In the study, some variables had extreme values, which caused the data distribution to deviate from the normal distribution. In line with this situation, non-parametric statistical methods were preferred instead of parametric tests. As a result of the power analysis, it was determined that the study should be conducted with at least 50 participants and the data collection process was completed with 58 individuals. Since the sample size was close to the targeted number, extreme values were not excluded from the analysis and all data were included in the statistical evaluation. A baseline imbalance in C-peptide levels was observed between groups. Although ANCOVA adjustment indicated that baseline C-peptide was not an independent predictor of 6-month HbA1c, residual confounding cannot be completely excluded.

The study was conducted as an open-label trial, as blinding was not feasible due to the educational nature of the intervention, which may have introduced performance bias. Although participants were randomly assigned using a web-based random number generator, allocation concealment was limited. The lack of blinding may have introduced performance or detection bias. However, outcomes such as HbA1c and handgrip were objective measurements, which reduces the likelihood of substantial measurement bias. In addition, the analyses were performed according to the per-protocol principle. Although no significant baseline differences were observed between completers and dropouts, excluding participants lost to follow-up may have slightly overestimated the intervention effects.

The low average age of the participants and the high average BMI made it difficult to diagnose sarcopenia in terms of muscle mass. However, evaluating muscle strength and physical performance together provided a more accurate assessment. Nevertheless, evaluation of sarcopenia with a larger sample size is necessary for the correct estimation of sarcopenia prevalence in this patient group. Although dietary protein intake was assessed throughout the study, modifying protein intake was beyond the scope of the intervention, which focused exclusively on advanced carbohydrate counting education. Therefore, the potential influence of optimized protein intake on sarcopenia-related outcomes could not be evaluated. Future studies specifically targeting muscle health may benefit from combining carbohydrate counting education with predefined protein intake goals and structured exercise interventions.

Finally, adherence to the training intervention was not quantitatively assessed using objective compliance measures, which may limit interpretation of intervention fidelity. Larger, blinded, and adequately powered studies with standardized diagnostic criteria and adherence monitoring are needed to confirm these findings.

## Supplementary Information

Supplementary Material 1

## Data Availability

No datasets were generated or analysed during the current study.
