# Supplementary material for: Effects of carbohydrate counting training versus standard nutritional and medical therapy on glycemic control and sarcopenia in type 2 diabetes: an open-label randomized controlled trial
Source: Diabetol Metab Syndr. 2026 Aug 18;18:249. doi: 10.1186/s13098-026-02258-1 (PMC13757229; doi:10.1186/s13098-026-02258-1)
Supplement: Supplementary file 1 [file 13098_2026_2258_MOESM1_ESM.docx]

**Supplemental Material:**

**Table S1:** Changes in participants’ energy and nutrients intake over time

| **Variable** | **Groups** | **Baseline** | **Week 12** | |  | **Week 24** | |  |  |  |
| --- | --- | --- | --- | --- | --- | --- | --- | --- | --- | --- |
|  |  |  |  | **p^d^** | ***η²*** |  | **p^e^** | ***η²*** | **p^f^** | ***η²*** |
| **Energy (kcal)** | Training | 1696.17±538 | 1600.34±436.16 | 0.237 | 0.046 | 1533.73±417.62 | 0.032 | 0.115 | 0.236 | 0.053 |
|  | Control | 1431.22±396.83 | 1334,81±367.56 | 0.158 | 0.057 | 1455.41±420.86 | 0.715 | 0.009 | 0.311 | 0.053 |
|  | p (*η²*) | 0.062 (0.073) | **0.020^c^ (0.100)** | 0.076 | 0.025 | 0.469 (0.009) | 0.173 | 0.025 | 0.258 | 0.028 |
| **Carbohydrate (g)** | Training | 149.9±52.8 | 143.41±50.53 | 0.178 | 0.018 | 131.41±50.62 | 0.116 | 0.109 | 0.355 | 0.038 |
|  | Control | 129.55±52.47 | 119.51±51.71 | 0.339 | 0.036 | 130.24±53.57 | 0.230 | 0.019 | 0.089 | 0.110 |
|  | p (*η²*) | 0.115 (0.037) | **0.029^c^ (0.053)** | 0.122 | 0.017 | 0.794 (0.000) | 0.597 | 0.008 | 0.235 | 0.030 |
| **Carbohydrate (%)** | Training | 37.06±9.60 | 35.93±6.02 | 0.770 | 0.025 | 34.70±7.31 | 0.797 | 0.029 | 0.300 | 0.045 |
|  | Control | 36.29±10.76 | 36.18±9.06 | 0.280 | 0.000 | 36.31±7.33 | 0.493 | 0.026 | 0.406 | 0.041 |
|  | p (*η²*) | 0.645 (0.001) | 0.755 (0.002) | 0.645 | 0.002 | 0.615 (0.012) | 0.749 | 0.015 | 0.776 | 0.001 |
| **Protein (g)** | Training | 67.68±20.94 | 71.37±25.46 | 0.368 | 0.027 | 65.61±21.42 | 0.752 | 0.004 | 0.462 | 0.061 |
|  | Control | 54.54±17.18 | 56.00±21.63 | 0.736 | 0.004 | 59.45±20.53 | 0.208 | 0.074 | 0.552 | 0.064 |
|  | p (*η²*) | **0.012^c^ (0.107)** | **0.018^c^ (0.098)** | 0.361 | 0.005 | 0.313 (0.022) | 0.642 | 0.003 | 0.552^b^ | 0.014 |
| **Protein (%)** | Training | 16.0[15-18.5] | 17.0[16.0-20.0] | 0.053 | 0.254 | 18.0[15.0-20.0] | 0.185 | 0.174 | 0.288 | 0.046 |
|  | Control | 16.5[13.0-18.0] | 17.0[15.0-18.0] | 0.257 | 0.148 | 17.0[15.0-18.0] | 0.457 | 0.097 | 0.555 | 0.027 |
|  | p (*η²*) | 0.531 (0.082) | 0.111 (0.209) | **0.001^g^** | **0.299** | 0.249 (0.151) | 0.133 | 0.197 | 0.232 | 0.030 |
| **Fat (g)** | Training | 85.3[67.4-108.0] | 75.0[64.0-93.2] | 0.280 | 0.141 | 80.8[71.0-96.3] | 0.055 | 0.252 | 0.169 | 0.066 |
|  | Control | 70.45[56.9-86.4] | 67.0[55.4-78.9] | 0.080 | 0.229 | 72.9[58.6-90.7] | 0.911 | 0.014 | 0.464 | 0.035 |
|  | p (*η²*) | 0.163 (0.183) | **0.015^c^ (0.317)** | 0.558 | 0.238 | 0.382 (0.114) | 0.110 | 0.209 | 0.122 | 0.043 |
| **Fat (%)** | Training | 46.19±9.56 | 46.03±6.34 | 0.294 | 0.038 | 47.63±7.22 | 0.899 | 0.001 | 0.344 | 0.040 |
|  | Control | 47.96±9.24 | 46.78±8.07 | 0.342 | 0.017 | 47.04±6.27 | 0.243 | 0.076 | 0.789 | 0.011 |
|  | p (*η²*) | 0.463 (0.009) | 0.203 (0.003) | 0.023 | 0.003 | 0.755 (0.002) | 0.516 | 0.002 | 0.652 | 0.009 |
| **Fiber (g)** | Training | 25.56±12.28 | 24.56±10.98 | 0.558 | 0.012 | 22.12±9.36 | **0.007^g^** | **0.270** | **0.009^c^** | **0.174** |
|  | Control | 20.25±8.50 | 19.31±6.03 | 0.382 | 0.020 | 20.50±8.11 | 0.768 | 0.001 | 0.334 | 0.050 |
|  | p (*η²*) | 0.134 (0.060) | 0.060 (0.081) | 0.339 | 0.085 | 0.644 (0.009) | 0.047 | 0.011 | 0.349 | 0.021 |
| **Saturated Fatty Acids (g)** | Training | 28.0[21.0-42.2] | 25.4[20.4-31.6] | 0.401 | 0.083 | 23.5[18.2-33.4] | 0.130 | 0.269 | 0.322 | 0.073 |
|  | Control | 21.4[18.1-27.6] | 18.7[15.5-25.7] | 0.059 | 0.252 | 23.7[19.5-26.5] | 0.919 | 0.022 | 0.056 | 0.072 |
|  | p (*η²*) | 0.131 (0.193) | **0.012^c^ (0.363)** | 0.076 | 0.201 | 0.693 (0.047) | 0.251 | 0.193 | 0.204^c^ | 0.033 |
| **Monounsaturated Fatty Acids (g)** | Training | 30.2[24.4-43.7] | 28.4[22.9-35.7] | 0.139 | 0.194 | 28.8[26.0-38.4] | 0.746 | 0.191 | 0.163 | 0.067 |
|  | Control | 26.7[21.3-38.1] | 24.5[19.9-32.1] | 0.166 | 0.181 | 27.2[21.8-34.7] | 0.297 | 0.136 | 0.201 | 0.073 |
|  | p (*η²*) | 0.450 (0.150) | 0.371 (0.229) | 0.036 | 0.275 | 0.794 (0.105) | 0.685 | 0.230 | **0.048^c^** | **0.062** |
| **Polyunsaturated Fatty Acids (g)** | Training | 12.23±9.01 | 18.24±7.73 | 0.619 | 0.001 | 18.81±8.01 | 0.146 | 0.004 | 0.555 | 0.022 |
|  | Control | 18.37±11.83 | 15.95±5.83 | 0.572 | 0.042 | 17.56±7.60 | 0.808 | 0.008 | 0.965 | 0.002 |
|  | p (*η²*) | 0.252 (0.000) | 0.080 (0.028) | 0.947 | 0.001 | 0.421 (0.028) | 0.079 | 0.001 | 0.613 | 0.010 |
| **Omega-3 Fatty Acids (g)** | Training | 1.5[1.0-2.0] | 1.3[1.2-1.6] | 0.374 | 0.117 | 1.3[1.1-1.6] | 0.220 | 0.161 | 0.428 | 0.031 |
|  | Control | 1.2[1.0-1.8] | 1.3[0.9-1.8] | 0.407 | 0.109 | 1.4[1.2-1.8] | 0.794 | 0.034 | 0.439 | 0.037 |
|  | p (*η²*) | 0.601 (0.068) | 0.558 (0.076) | 0.232 | 0.156 | 0.419 (0.106) | 0.475 | 0.094 | 0.928 | 0.002 |
| **Omega-6 Fatty Acids (g)** | Training | 16.5±8.25 | 16.91±7.85 | 0.516 | 0.003 | 17.50±8.14 | 0.810 | 0.013 | 0.972 | 0.001 |
|  | Control | 16.60±11.29 | 14.64±6.04 | 0.726 | 0.029 | 15.72±6.75 | 0.881 | 0.011 | 0.988 | 0.001 |
|  | p (*η²*) | 0.400 (0.000) | 0.314 (0.026) | 0.790 | 0.004 | 0.608 (0.014) | 0.755 | 0.028 | 0.995 | 0.000 |
| **Cholesterol (mg)** | Training | 291.91±129.21 | 317.32±113.57 | 0.280 | 0.044 | 299.97±116.71 | 0.719 | 0.001 | 0.717 | 0.012 |
|  | Control | 258.36±180.84 | 289.49±170.31 | 0.109 | 0.031 | 294.79±131.94 | 0.244 | 0.021 | 0.211 | 0.017 |
|  | p (*η²*) | 0.129 (0.012) | **0.046^c^ (0.010)** | 0.050 | 0.036 | 0.998 (0.000) | 0.515 | 0.006 | 0.463 | 0.016 |

^a^ For the significance of the data in this table, Mann Whitney *U*, Independent Samples *t*-test, Wilcoxon, Dependent Samples *t*-test and Friedman test were applied. ^b^ Mixed Design ANOVA method was used in this data analysis. ^c^ The accepted level for significance is p<0.05. ^d^ Significance analysis of paired assessment of baseline and week 12 measurements ^e^ Significance analysis of paired assessment of baseline and week 24 measurements ^f^ Significance analysis of triple assessment of baseline, week 12 and week 24 measurements ^g^ Bonferroni correction was applied for Post Hoc Analyses: p<0.017. ^h^ Mean ± SD values have been provided for normally distributed (parametric) variables and Median [IQR] values for non-parametric variables.  ^i^ Effect sizes are presented as *η²*, *r*, partial *η²*, or Kendall’s *W* depending on the statistical test applied.

**Table S2:** Content of the Advanced Carbohydrate Counting Training.

| **Topics** | **Content** |
| --- | --- |
| **Day 1: Basic Carbohydrate Counting Training (2 sessions- Total 90 min)** | |
| 1. Principles of medical nutrition therapy in diabetes | - Daily energy requirement and distribution of nutrients - Frequency of meals and number of meals - Use of diabetic and light products |
| 2. Carbohydrates | - Content of macronutrients in food groups - Effects of macronutrients on plasma glucose - Glycaemic index/glycaemic load - Meal planning with healthy food choices |
| 3. Insulin Metabolism and Diabetes | - Basal and prandial insulin production and metabolism - The effects of insulin resistance and insulin deficiency on the body in diabetes |
| 4. Hypoglycaemia | - Symptoms of Hypoglycemia - Treatment of Hypoglycemia - Prevention of Hypoglycemia |
| 5. Carbohydrates Counting | - Carbohydrate amounts in foods - Portions of foods containing 15 grams of carbohydrate defined as 1 carbohydrate change |
| 6. Practical Methods of Carbohydrates Counting | - Calculating carbohydrates in grams, - Calculating the average carbohydrate levels of food group portions |
| 7. Home Assignment | - Practice at home and use of worksheets for next training session |
| **Day 2:Intermediate Carbohydrate Counting Training (1 session- 45 min)** | |
| 8. Brief review of previous training | - Effects of carbohydrates on blood glucose, glycemic index and importance of food selection |
| 9. Practice calculating the amount of carbohydrates in a meal | - Using a 3-day food consumption record, patients calculated the amount of carbohydrate they consumed at all meals during the day. |
| 10. Detection of conditions affecting plasma glucose | - Effects of physical activity, illness, stress, alcohol use on plasma glucose. - Insulin use in physical activity, illness, stress, alcohol use. |
| 11. Home Assignment | - Practice at home and use of worksheets for next training session |
| **Day 3: Advanced Carbohydrate Counting Training (1 session- 45 min)** | |
| 12. Practice calculating the amount of carbohydrates in a meal | - Using a 3-day food consumption record, patients calculated the amount of carbohydrate they consumed at all meals during the day. |
| 13. Detection of conditions affecting plasma glucose | - Effects of physical activity, illness, stress, alcohol use on plasma glucose. - Insulin use in physical activity, illness, stress, alcohol use. |
| 14. Insulin Correction Factor and Carbohydrate/Insulin Ratio | - Calculation of insulin correction factor and carbohydrate/insulin ratio - Adjusting insulin dose according to individual differences in daily life |

**Figure S1:** Worksheet of Advanced Carbohydrate Counting Training/ Food Consumption Record
